# Amelioratory Effect of Nanoconjugated Vancomycin on Spleen during VRSA-Induced Oxidative Stress

**DOI:** 10.4061/2011/420198

**Published:** 2011-07-20

**Authors:** Subhankari Prasad Chakraborty, Santanu KarMahapatra, Sumanta Kumar Sahu, Panchanan Pramanik, Somenath Roy

**Affiliations:** ^1^Immunology and Microbiology Laboratory, Department of Human Physiology with Community Health, Vidyasagar University, West Bengal, Midnapore 721 102, India; ^2^Nanomaterials Laboratory, Department of Chemistry, Indian Institute of Technology, Kharagpur, West Bengal, Kharagpur 721 302, India

## Abstract

*Objective*. The aim of the present study was to evaluate the possible antioxidant effects of nanoconjugated vancomycin against VRSA infection on select makers of oxidative damage and antioxidant status in spleen. *Methods*. A coagulase-positive VRSA strain was used for this study. VRSA infection was developed in Swiss mice by intraperitoneal injection of 5 × 10^6^ CFU/mL bacterial solutions. VRSA-infected mice were treated with nanoconjugated vancomycin at its effective dose for 10 days. After decapitation, blood was used for determination of viable bacteria count and spleen was excised from control and experimental groups, homogenized and used for different biochemical estimations. *Results*. Nitrate level, myeloperoxidase activity, lipid peroxidation, protein oxidation, oxidized glutathione, and DNA fragmentation level were increased significantly (*P* < 0.05) in spleen of VRSA-infected group as compared to control group, and reduced glutathione level, activity of SOD, CAT, GPx, GR, and GST were decreased significantly (*P* < 0.05); which were increased or decreased significantly (*P* < 0.05) near to normal in nanoconjugated vancomycin-treated group. *Conclusion*. These findings suggest the potential use and beneficial role of nanoconjugated vancomycin against VRSA-infection-induced oxidative stress and DNA damage in spleen.

## 1. Introduction

*Staphylococcus aureus* is facultatively anaerobic, Gram-positive coccus and is the most common cause of staph infections. It is frequently part of the skin flora found in the nose and on skin. About 20% of the human populations are long-term carriers of *S. aureus* [1]. *S. aureus *has developed resistance to most classes of antimicrobial agents. Penicillin was the first choice of antibiotics to treat staphylococcal infection. In 1944, by destroying the penicillin by *penicillinase*, *S. aureus* became resistant [2]. More than 90% of *S. aureus *strains are resistant to penicillin [3]. Methicillin, a semisynthetic penicillin, was used to treat penicillin-resistant *Staphylococcus aureus* but resistance finally emerged in 1962 [4, 5]. Vancomycin, a glycopeptide antibiotic, continues to be an important antimicrobial agent to treat MRSA but resistance finally emerges. In June 2002, the world's first reported clinical infection due to *S. aureus* with high resistance to vancomycin (VRSA) was diagnosed in a patient in the USA [6]. Recently, we have isolated thirty pathogenic *S. aureus* from post-operative pus sample; out of them twenty-two were vancomycin sensitive and rests were vancomycin resistant [7]. 

*S*. *aureus* causes chronic or relapsing diseases and is reported to persist as an opportunistic intracellular organism both in vitro and in vivo [8]. *S. aureus *was able to survive within phagocytic cells both in polymorphonuclear leukocytes (PMN) and monocytes [9]. In vitro studies demonstrated that pathogenic strains of *S. aureus *could survive for long periods of time inside both PMN and monocytes isolated from different animals and humans. To induce an infection in host, the pathogens must cope with their changing environment and have to attack continuously the host to weaken the immune system [10]. 

Chitin is the major structural component of invertebrates like crab, shrimp, shells and the cell walls of fungi. Chitosan (CS), the deacetylated form of chitin, is a linear polysaccharide, composed of glucosamine and N-acetyl glucosamine linked in a *β*-linkage [11]. CS has been reported to possess immune stimulating properties such as increasing accumulation and activation of macrophages and polymorphonucleus, augmenting antibody responses and inducing production of cytokines [12]. Carboxymethyl chitosan (CMC) is a linear polysaccharide composed of *β* (1,4) glycosidic linkages between 6-carboxymethyl-D-glucosamine monomers. CMC is synthesized from CS by carboxylation of the hydroxyl and amine groups [13]. In our previous laboratory report, we synthesized CMC-EDBE-FA nanoparticle based on carboxy methyl chitosan tagged with folic acid by covalent linkage through 2,2′-(ethylenedioxy)bis(ethylamine), vancomycin was loaded onto it, the complex is called “nanoconjugated vancomycin,” observed its bactericidal activity against *S. aureus* [14]; and reported that CMC-EDBE-FA nanoparticle is nontoxic [15]. We also reported that in vivo challenge of VSSA and VRSA for 5 days can produce the highest degree of damage in lymphocyte through the increased production of nitric oxide, and TNF-*α* that leads to decreased antioxidant status in cell and 10 days of successive treatment of nanoconjugated vancomycin also eliminate in vivo VSSA and VRSA infection [16]. In light of these findings, the present study was conducted to obtain information on the possible antioxidant effects of nanoconjugated vancomycin against VRSA infection on select makers of oxidative damage and antioxidant status in spleen. 

## 2. Materials and Methods

### 2.1. Chemicals and Reagents

Sodium dodecyl sulfate (SDS), 5′,5′-dithio(bis)-2-nitrobenzoic acid (DTNB), standard reduced glutathione (GSH), glutathione reductase (GR), NADPH, Na_4_, NADPH, and oxidized glutathione (GSSG) were purchased from Sigma Chemical Co., USA. Sodium chloride (Nacl), sodium dodecyl sulfate, sucrose, ethylene diamine tetra acetate (EDTA), tryptic soy broth, and mannitol salt agar were purchased from Himedia, India. Tris-Hcl, KH_2_PO_4_ and K_2_HPO_4_, alcohol, formaldehyde, paraffin wax, xylene, haematoxylin, eosin, DPX, diphenylamine (DPA), O-phenylenediamine, and other chemicals were procured from Merck Ltd., SRL Pvt. Ltd., Mumbai, India. All other chemicals were from Merck Ltd., SRL Pvt., Ltd., Mumbai and were of the highest grade available. 

### 2.2. Animals

Experiments were performed using twenty-four (24) Swiss male mice, 6–8 weeks old, weighing 20–25 g. The animals were fed standard pellet diet and were given water* ad libitum* and housed in polypropylene cage (Tarson) in the departmental animal house with 12 h light : dark cycle and the temperature of 25 ± 2°C. The animals were allowed to acclimatize for one week. The animals used did not show any sign of malignancy or other pathological processes. Animals were maintained in accordance with the guidelines of the National Institute of Nutrition, Indian Council of Medical Research, Hyderabad, India, and approved by the ethical committee of Vidyasagar University. 

### 2.3. Bacterial Strain

We used a coagulase-positive vancomycin-resistant *Staphylococcus aureus *(MMC-17) strain that was isolated from human post-operative pus sample and was grown at 37°C for overnight in tryptic soy broth [7]. The bacterial culture was centrifuged at 15,000 rpm for 15 minutes. The pellet was resuspended and washed with sterile phosphate buffer saline (PBS). Using a UV spectrophotometer (Shimadzu, USA) at an absorbance of 620 nm, we adjusted the viable bacterial count to approximately 1.0 × 10^9^ colony-forming units (CFU)/mL, which corresponded to an optical density of 1.6. The bacterial suspension was adjusted by serial dilution in PBS to give a final concentration of approximately 5 × 10^6^ in 100 *μ*L of bacterial suspension [17]. 

### 2.4. Preparation of CMC-EDBE-FA Nanoparticle and Loading of Vancomycin

CMC-EDBE-FA nanoparticle was prepared and vancomycin was loaded onto it according to our previous laboratory report [14]. 

### 2.5. Development of VRSA Infection in Swiss Mice

VRSA infection was developed in male Swiss mice by intraperitoneal (i.p.) injection of 100 *μ*L of bacterial suspension containing 5 × 10^6^ CFU/mL according to our previous laboratory report [16]. 

### 2.6. Experimental Design

VRSA-infected mice were treated with nanoconjugated vancomycin for successive 10 days at a dose of 500 mg/kg bw/day. The dose and duration of nanoconjugated vancomycin was selected from our previous laboratory report [16]. The following groups were considered for the experiment: Group I: control, Group II: VRSA infection, Group III: VRSA infection+500 mg/kg bw/day nanoconjugated vancomycin. After the termination of the experiment, animals were sacrificed by an intraperitoneal injection of sodium pentobarbital (60–70 mg/kg body weight) [18] and blood (*n* = 6/group) was used for the determination of viable bacteria count. 

### 2.7. Separation and Homogenization of Spleen

After decapitation, spleen was excised from experimental mice of different experimental groups and washed with cold normal saline. Washed tissues of two mice from each group were perfused with normal saline and formalin for histological study and the rest washed tissues of six mice were immediately homogenized in the ice-cold buffer containing 0.25 M sucrose, 1 mM EDTA, and 1 mM Tris-Hcl, pH 7.4. The homogenate was first centrifuged at 600 ×g for 10 min at 4°C, and the supernatant was stored at −80°C for the biochemical estimation of different parameters. 

### 2.8. Histopathological Evaluation

Histological analysis of spleen of each experimental group was performed by the method of Iranloye and Bolarinwa [19]. The tissues that were perfused in saline and formalin were fixed for 7 days in 10% formaldehyde after which dehydration was carried out in ascending grade of alcohol. The tissues were then cleared of xylene overnight (16 hours) to remove the alcohol. Infiltration/impregnation was done in three changes of molten soft paraffin wax at ≤68°C for 1 hour each. Embedding and casting in paraffin wax with wooden block was done and sectioning of 5 *μ*m thick carried out using a microtome. The sectioned tissues of spleen were mounted on slides using a thin film of egg albumen smeared on each side. The sections were deparaffinized in xylene, passed through alcohol, stained with haematoxylin-eosin, and mounted in neutral DPX medium. The slides were then evaluated for pathological changes under Olympus research phase contrast microscope (Model: CX41; Olympus Singapore Pvt. Ltd., Valley Point Office Tower, Singapore). 

### 2.9. Quantification of Viable Bacteria in Blood

100 *μ*L of blood was inoculated in 1.0 mL sterile tryptic soy broth with sterile disposable microtips, grown at 37°C for overnight, plated onto tryptic soy agar and mannitol salt agar plates in triplicate, and incubated at 37°C for 24 hours. Colonies were counted by dilution platting method and expressed as CFU per mL. 

### 2.10. Biochemical Estimation

#### 2.10.1. Nitrite (NO) Level

After treatment schedule, 100 *μ*L of Griess reagent (containing 1 part of 1% sulfanilamide in 5% phosphoric acid, and 1 part of 0.1% of N-C-1 naphthyl ethylene diamine dihydrochloride) was added to 100 *μ*L of sample, incubated at room temperature for 10 minutes; readings were taken in a UV spectrophotometer at 550 nm and compared to a sodium nitrite standard curve (values ranging between 0.5 and 25 *μ*M). The levels of NO were expressed as *μ*M/mg protein [16]. 

#### 2.10.2. Determination of Myeloperoxidase (MPO) Activity

200 *μ*L of cell lysate was reacted with 200 *μ*L substrate (containing H_2_O_2_ and OPD) in the dark for 30 min. The blank was prepared with citrate phosphate buffer (pH 5.2) and substrate, in absence of cell-free supernatant. The reaction was stopped with addition of 100 *μ*L 2(N) sulfuric acid, and reading was taken at 492 nm in a spectrophotometer [16]. The MPO activity was expressed in terms of *μ*M/mg protein. 

#### 2.10.3. Determination of Lipid Peroxidation (MDA) Level

Lipid peroxidation of spleen homogenate was estimated by the method of KarMahapatra et al., 2009 [20]. Briefly, the reaction mixture contained Tris-HCl buffer (50 mM, pH 7.4), tetra-butyl hydroperoxide (BHP) (500 *μ*M in ethanol), and 1 mM FeSO_4_. After incubating the samples at 37°C for 90 min, the reaction was stopped by adding 0.2 mL of 8% sodium dodecyl sulfate (SDS) followed by 1.5 mL of 20% acetic acid (pH 3.5). The amount of malondialdehyde (MDA) formed during incubation was estimated by adding 1.5 mL of 0.8% TBA and further heating the mixture at 95°C for 45 min. After cooling, samples were centrifuged, and the TBA reactive substances (TBARS) were measured in supernatants at 532 nm by using 1.53 × 10^5^ M^−1^ cm^−1^ as extinction coefficient. The levels of lipid peroxidation were expressed in terms of nmol/mg protein. 

#### 2.10.4. Determination of Protein Carbonyl (PC) Contents

Protein oxidation was monitored by measuring protein carbonyl contents by derivatization with 2,4-dinitrophenyl hydrazine (DNPH) [20]. In general, spleen proteins in 50 mM potassium phosphate buffer, pH 7.4, were derivatized with DNPH (21% in 2 N HCl). Blank samples were mixed with 2 N HCl incubated at 1 h in the dark; protein was precipitated with 20% trichloroacetic acid (TCA). Underivatized proteins were washed with an ethanol : ethyl acetate mixture (1 : 1). Final pellets of protein were dissolved in 6 N guanidine hydrochloride, and absorbance was measured at 370 nm. Protein carbonyl content was expressed in terms of nmol/mg protein. 

#### 2.10.5. Determination of Reduced Glutathione (GSH) Level

Reduced glutathione estimation in spleen homogenate was performed by the method of KarMahapatra et al., 2009 [20]. The required amount of sample was mixed with 25% of TCA and centrifuged at 2,000 ×g for 15 min to settle the precipitated proteins. The supernatant was aspirated and diluted to 1 mL with 0.2 M sodium phosphate buffer (pH 8.0). Later, 2 mL of 0.6 mM DTNB was added. After 10 minutes the optical density of the yellow-colored complex formed by the reaction of GSH and DTNB (Ellman's reagent) was measured at 405 nm. A standard curve was obtained with standard reduced glutathione. The levels of GSH were expressed as *μ*g of GSH/mg protein. 

#### 2.10.6. Determination of Oxidized Glutathione (GSSG) Level

The oxidized glutathione level in spleen homogenate was measured after derivatization of GSH with 2-vinylpyidine according to the method of KarMahapatra et al., 2009 [20]. In brief, with 0.5 mL sample, 2 *μ*L of 2-vinylpyidine was added and incubated for 1 hr at 37°C. Then the mixture was deprotenized with 4% sulfosalicylic acid and centrifuged at 1,000 ×g for 10 min to settle the precipitated proteins. The supernatant was aspirated and GSSG level was estimated with the reaction of DTNB at 412 nm in spectrophotometer and calculated with standard GSSG curve. The levels of GSSG were expressed as *μ*g of GSSG/mg protein.

#### 2.10.7. Determination of Super Oxide Dismutase (SOD) Activity

SOD activity of spleen homogenate was determined from its ability to inhibit the auto-oxidation of pyrogallol according to KarMahapatra et al., 2009 [20]. The reaction mixture considered consisted of 50 mM Tris (hydroxymethyl) amino methane (pH 8.2), 1 mM diethylenetriamine penta acetic acid, and 20–50 *μ*L of sample. The reaction was initiated by addition of 0.2 mM pyrogallol and the absorbance measured kinetically at 420 nm at 25°C for 3 min. SOD activity was expressed as unit/mg protein. 

#### 2.10.8. Determination of Catalase (CAT) Activity

Catalase activity of spleen homogenate was measured by the method of KarMahapatra et al., 2009 [20]. The final reaction volume of 3 mL contained 0.05 M Tris-buffer, 5 mM EDTA (pH 7.0), and 10 mM H_2_O_2_ (in 0.1 M potassium phosphate buffer, pH 7.0). About 50 *μ*L of sample was added to the above mixture. The rate of change of absorbance per min at 240 nm was recorded. Catalase activity was calculated by using the molar extinction coefficient of 43.6 M^−1^ cm^−1^ for H_2_O_2_. The level of CAT was expressed as unit/mg protein. 

#### 2.10.9. Determination of Glutathione Peroxidase (GPx) Activity

The GPx activity of spleen homogenate was measured by the method of KarMahapatra et al., 2009 [20]. The reaction mixture contained 50 mM potassium phosphate buffer (pH 7.0), 1 mM EDTA, 1 mM sodium azide, 0.2 mM NADPH, 1 U glutathione reductase, and 1 mM reduced glutathione. The sample, after its addition, was allowed to equilibrate for 5 min at 25°C. The reaction was initiated by adding 0.1 mL of 2.5 mM H_2_O_2_. Absorbance at 340 nm was recorded for 5 min. Values were expressed as nmol of NADPH oxidized to NADP by using the extinction coefficient of 6.2 × 10^3^ M^−1^ cm^−1^ at 340 nm. The activity of GPx was expressed in terms of nmol NADPH consumed/min/mg protein. 

#### 2.10.10. Determination of Glutathione Reductase (GR) Activity

The GR activity spleen homogenate was measured by the method of KarMahapatra et al., 2009 [20]. The tubes for enzyme assay were incubated at 37°C and contained 2.0 mL of 9 mM GSSG, 0.02 mL of 12 mM NADPH, Na4, 2.68 mL of 1/15 M phosphate buffer (pH 6.6), and 0.1 mL of sample. The activity of this enzyme was determined by monitoring the decrease in absorbance at 340 nm. The activity of GR was expressed in terms of nmol NADPH consumed/min/mg protein. 

#### 2.10.11. Determination of Glutathione-S-Transferase (GST) Activity

The GST activity of spleen homogenate was measured by the method of KarMahapatra et al., 2009 [20]. The tubes of enzyme assay were incubated at 25°C and contained 2.85 mL of 0.1 M potassium phosphate (pH 6.5) containing 1 mM of GSH, 0.05 mL of 60 mM 1-chloro-2, 4-dinitrobengene and 0.1 mL of sample. The activity of this enzyme was determined by monitoring the increase in absorbance at 340 nm. The activity of GST was expressed in terms of nmol NADPH consumed/min/mg protein. 

#### 2.10.12. DNA Fragmentation Assay by Diphenylamine (DPA) Assay

The diphenylamine (DPA) reaction of spleen was performed by the method of Perandones et al., 1993 [21]. Perchloric acid (0.5 M) was added to the sample containing uncut DNA (resuspended in 200 *μ*L of hypotonic lysis buffer) and to the other half of the supernatant containing DNA fragments. Then two volumes of a solution consisting of 0.088 M DPA, 98% (v/v) glacial acetic acid, 1.5% (v/v) sulphuric acid, and a 0.5% (v/v) concentration of 1.6% acetaldehyde solution were added. The samples were stored at 4°C for 48 h. The reaction was quantified spectrophotometrically at 575 nm. The percentage of fragmentation was calculated as the ratio of DNA in the supernatants to the total DNA. 

#### 2.10.13. Protein Estimation

Protein was determined using bovine serum albumin as standard according to Lowry et al., 1951 [22].

### 2.11. Statistical Analysis

The data were expressed as mean ± SEM, *n* = 6. Comparisons between the means of control and VRSA-treated group were made by two-way ANOVA test (using a statistical package, Origin 6.1, Northampton, Mass, USA) with multiple comparison *t*-tests, *P* < 0.05 as a limit of significance. 

## 3. Results

### 3.1. Characterization of CMC-EDBE-FA

The peak assignment of CMC was as follows: 1741 cm^−1^ (–COOH), 1070–1136 cm^−1^ (–C–O) and 1624 and 1506 cm^−1^ (–NH_3_^+^). FA-EDBE showed the characteristic absorption bands at 1650 and 1550 cm^−1^ located in the zone related to the (–CONH–), corresponding, respectively, to the (C=O) stretching band and to the (–NH) bending vibration band. The presence of these two bands indicates that an amide bond has been formed between –COOH of folic acid and the –NH_2_ amine end group of EDBE. More characteristics of these two bands have become more prominent and intense in CMC-EDBE-FA. This provides evidence for the formation of an extra amide bond during the attachment of folic acid. ^1^H NMR spectrum of CMC-EDBE-FA showed the peaks at about 1.9 ppm attributed to the methyl hydrogen of acetamido-2-deoxy-*β*-D-glucopyranosyl unit; the peaks at about 2.9–3.2 ppm attributed to methylene hydrogen atoms of EDBE and 3.5–4 ppm observed the glucopyranosyl hydrogen atoms. It was clear that the proton peaks of 8.7, 7.6, 6.9, and 6.4 ppm were observed in ^1^H NMR spectrum of CMC-EDBE-FA. No such peaks were observed in the same chemical shifts of ^1^H NMR spectrum for CMC. The appearance of these peaks confirms the successful conjugation of FA-EDBE with CMC. The size of CMC-EDBE-FA self-assembled nanoparticles in aqueous medium measured by dynamic laser light scattering (DLS) ranged from 210 ± 40 nm. The morphology of CMC-EDBE-FA self-aggregated nanoparticles was investigated by TEM. The nanoaggregate shows a spherical geometry and has a uniform size. At lower magnification nanoparticles having an average size of about 50 nm were observed (figures are not shown) [14]. 

### 3.2. Histopathological Study

**Figure 1:**
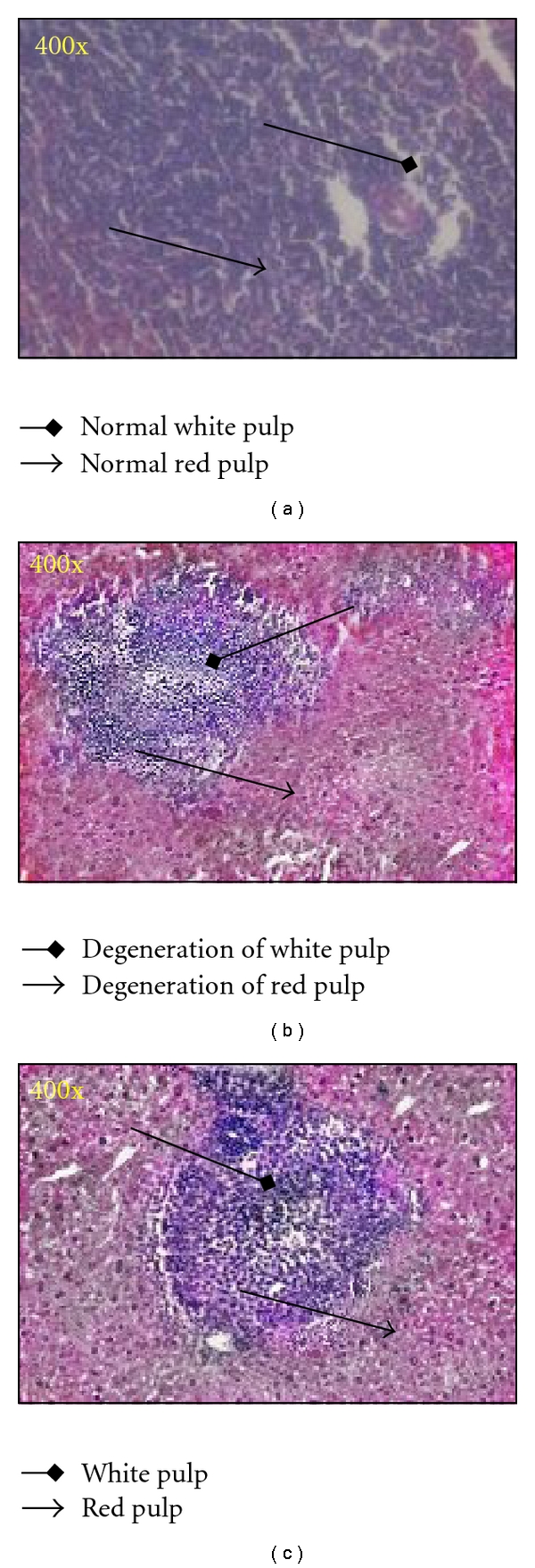
Photomicrographs of the histopathological analysis of the mice spleen tissues (400x). (a) Control group: normal red pulp (arrow) and white pulp (black diamond); (b) VRSA-infected group: degeneration of red pulp (arrow) and white pulp (black diamond); (c) nanoconjugated-vancomycin-treated group: red pulp (arrow) and white pulp (black diamond).

Histopathological analysis revealed that VRSA infection resulted in severe pathological changes and tissue injury in spleen. On the other hand, treatment of nanoconjugated vancomycin showed recovery of tissue damage in spleen. This was confirmed by histopathological examinations of the spleen. Microscopic observations of control spleen showed regular structure of red pulp and white pulp (Figure 1(a)) while in VRSA-infected group the spleen showed degeneration of red pulp and white pulp (Figure 1(b)). Treatment of nanoconjugated vancomycin in VRSA-infection group caused regeneration of red pulp (Figure 1(c)). 

### 3.3. Viable Bacteria Count in Blood

**Figure 2:**
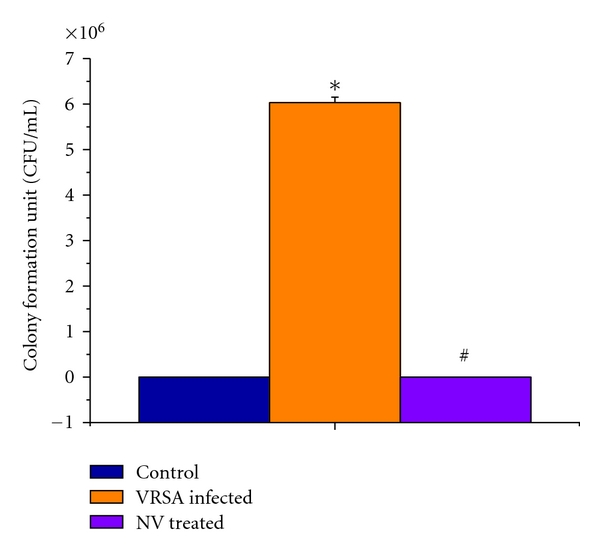
Viable bacteria count in blood of control, VRSA-infected, and nanoconjugated-vancomycin-treated group. Values are expressed as mean ± SEM, *n* = 6. *indicates significant difference (*P* < 0.05) compared to control group. ^#^indicates significant difference (*P* < 0.05) compared to VRSA-infected group.

From our study it was observed that viable bacterial count in blood was increased significantly in VRSA-infected group which was eliminated after treatment with nanoconjugated vancomycin (Figure 2). 

### 3.4. Nitrite (NO) Level and Myeloperoxidase (MPO) Activity

**Figure 3:**
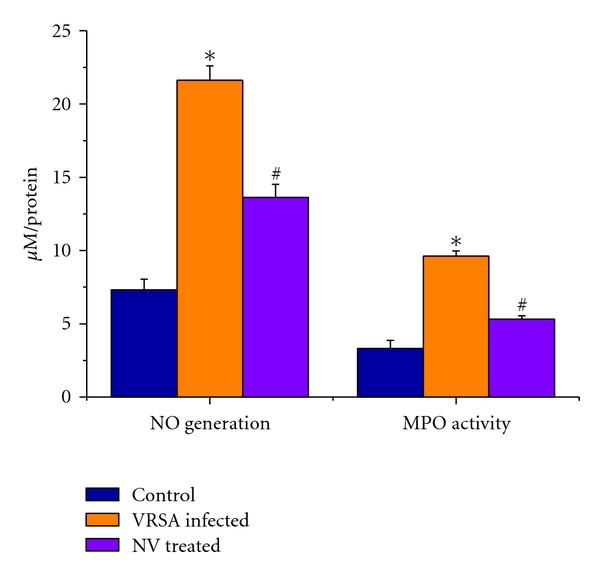
Nitrate (NO) generation and myeloperoxidse (MPO) in spleen of control, VRSA-infected, and nanoconjugated-vancomycin-treated group. Values are expressed as mean ± SEM, *n* = 6. *indicates significant difference (*P* < 0.05) compared to control group. ^#^indicates significant difference (*P* < 0.05) compared to VRSA-infected group.

Nitrate (NO) is an indicator of free radical generation. Myeloperoxidase (MPO) is an important enzyme to produce hypochlorous acid (HOCl) in cellular system that leads to oxidative damage. NO level and MPO activity were significantly (*P* < 0.05) increased in spleen by 195.49% and 190.94%, respectively, due to VRSA infection as compared to control group, in which they were significantly (*P* < 0.05) decreased by 37.03% and 44.76% due to treatment of nanoconjugated vancomycin (Figure 3). 

### 3.5. Lipid Peroxidation (MDA) and Protein Oxidation (PC) Level

**Figure 4:**
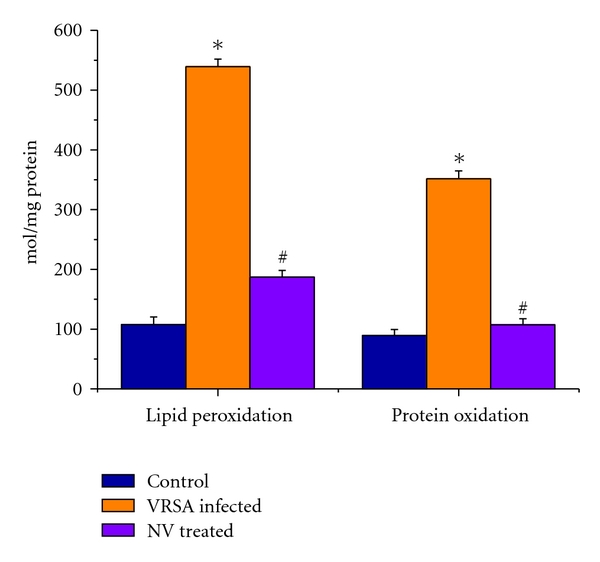
Lipid peroxidation (MDA) level and protein carbonyl (PC) contents in spleen of control, VRSA-infected, and nanoconjugated-vancomycin-treated group. Values are expressed as mean ± SEM, *n* = 6. *indicates significant difference (*P* < 0.05) compared to control group. ^#^indicates significant difference (*P* < 0.05) compared to VRSA-infected group.

Lipid peroxidation and protein oxidation are two important determinants to access the cellular damage. Lipid peroxidation in terms of malondialdehyde level and protein oxidation in terms of protein carbonyl level were significantly (*P* < 0.05) increased in spleen of VRSA-infected group by 399.90% and 172.36%, respectively, as compared to control group, in which they were significantly (*P* < 0.05) decreased by 65.28% and 69.54% due to treatment of nanoconjugated vancomycin (Figure 4). 

### 3.6. Reduced Glutathione (GSH) and Oxidized Glutathione (GSSG)

**Figure 5:**
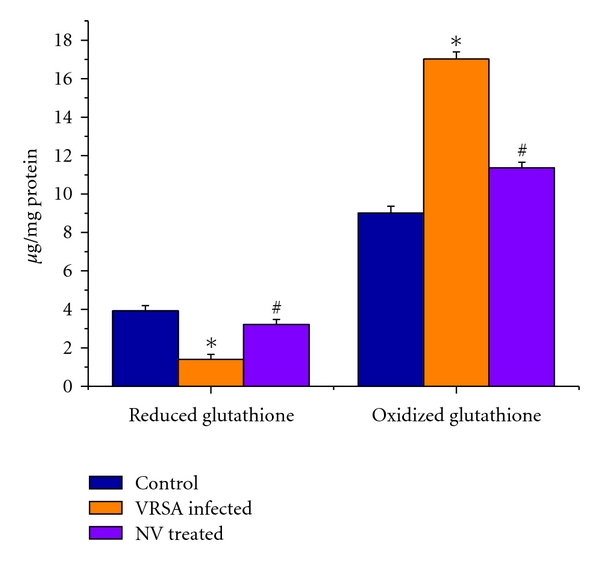
Reduced glutathione (GSH) and oxidized glutathione (GSSG) level in spleen of control, VRSA-infected, and nanoconjugated-vancomycin-treated group. Values are expressed as mean ± SEM, *n* = 6. *indicates significant difference (*P* < 0.05) compared to control group. ^#^indicates significant difference (*P* < 0.05) compared to VRSA-infected group.

Glutathione is an important antioxidant in cellular system. So, to understand glutathione level, we have measured both reduced and oxidized form of glutathione. The reduced glutathione level was decreased significantly (*P* < 0.05) by 64.46% in spleen of VRSA-infected group, as compared to control, whereas the oxidized glutathione level was increased significantly (*P* < 0.05) by 88.94%, as compared to control. Treatment of nanoconjugated vancomycin significantly (*P* < 0.05) increased GSH level by 130.65% and decreased GSSG level significantly (*P* < 0.05) by 33.28% in spleen (Figure 5). 

### 3.7. Superoxide Dismutase (SOD) and Catalase (CAT) Activity

**Figure 6:**
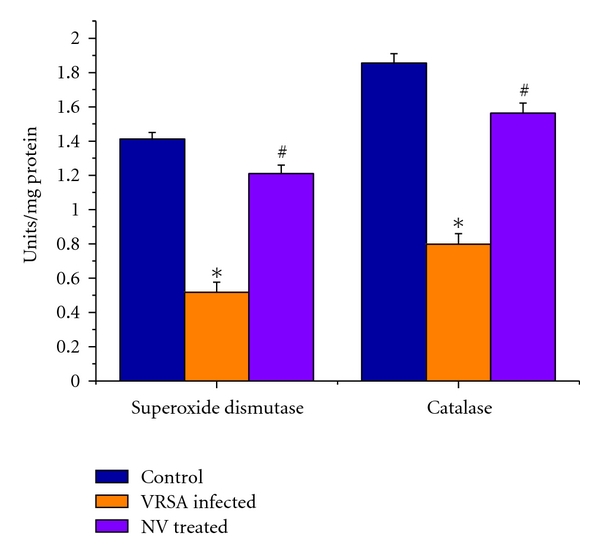
Superoxide dismutase (SOD) and catalase (CAT) activity in spleen of control, VRSA-infected, and nanoconjugated-vancomycin-treated group. Values are expressed as mean ± SEM, *n* = 6. *indicates significant difference (*P* < 0.05) compared to control group. ^#^indicates significant difference (*P* < 0.05) compared to VRSA-infected group.

The super oxide dismutase (SOD) and catalase (CAT) activities were measured to understand the antioxidant enzymes status in spleen of VRSA-infected group. SOD and CAT activities were decreased significantly (*P* < 0.05) by 63.31% and 56.95% in spleen of VRSA-infected group, respectively, as compared to control, in which they were significantly (*P* < 0.05) increased by 133.78% and 95.62% due to treatment of nanoconjugated vancomycin (Figure 6). 

### 3.8. Glutathione Peroxidase (GPx), Glutathione Reductase (GR), and Glutathione-S-Transferase (GST) Activity

**Figure 7:**
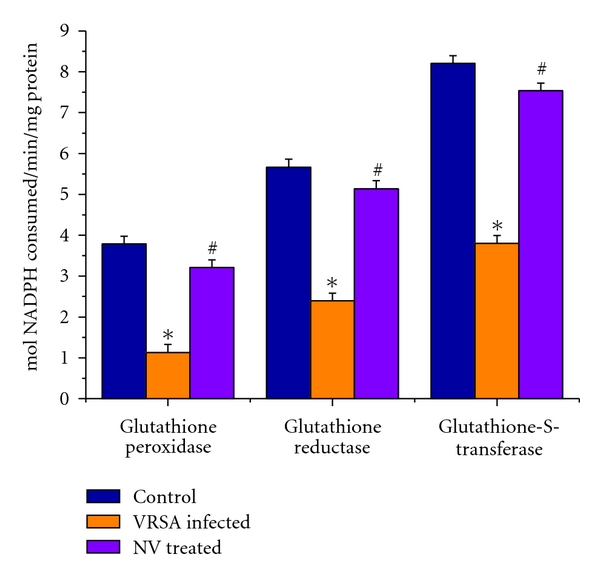
Glutathione peroxidase (GPx), glutathione reductase (GR), and glutathione-s-transferase (GST) activity in spleen of control, VRSA-infected, and nanoconjugated-vancomycin-treated group. Values are expressed as mean ± SEM, *n* = 6. *indicates significant difference (*P* < 0.05) compared to control group. ^#^indicates significant difference (*P* < 0.05) compared to VRSA-infected group.

Glutathione peroxidase (GPx), glutathione reductase (GR), and glutathione-s-transferase (GST) were measured to understand the antioxidant enzymes status in spleen of VRSA-infected group. GPx, GR, and GST activities were decreased significantly (*P* < 0.05) by 70.12%, 47.28%, and 53.68% in spleen of VRSA-infected group, respectively, as compared to control, in which they were significantly (*P* < 0.05) increased by 184.08%, 114.4%, and 98.26% due to treatment of nanoconjugated vancomycin (Figure 7). 

### 3.9. DNA Fragmentation

**Figure 8:**
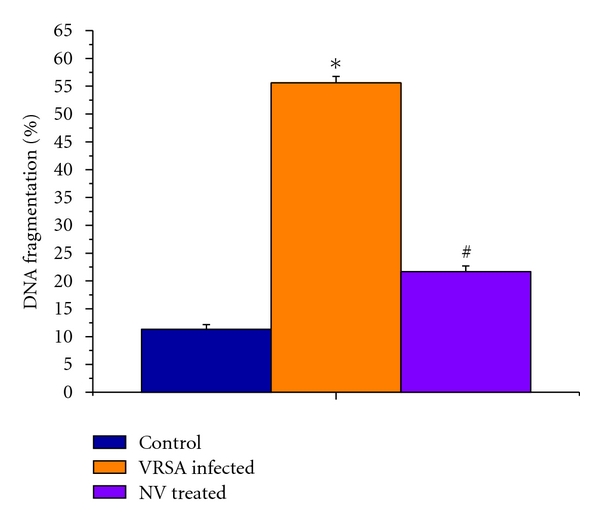
Quantitative estimation of DNA fragmentation assay by diphenylamine (DPA) assay in spleen of control, VRSA-infected, and nanoconjugated-vancomycin-treated group. Values are expressed as mean ± SEM, *n* = 6. *indicates significant difference (*P* < 0.05) compared to control group. ^#^indicates significant difference (*P* < 0.05) compared to VRSA-infected group.

DNA fragmentation was significantly (*P* < 0.05) increased in spleen by 391.43% as compared to control in which it was significantly (*P* < 0.05) decreased by 61.01% due to treatment of nanoconjugated vancomycin (Figure 8). 

## 4. Discussion

Staphylococcal infections caused by VRSA are increasing and their reduced therapeutic responsiveness to vancomycin represents an emerging threat to public health. Infection is a major complication of implanted devices. Patients frequently do not respond to high doses of antimicrobial agents even if administered for prolonged periods, and foreign bodies usually must be removed to achieve cure. It is known that susceptibility to antibiotics may be profoundly affected by growth of microorganisms near biomaterials. In vivo models are characterized by an artificial environment in which bacteria grow for a short time in the absence of host factors under selected, not necessarily physiologic, conditions.

The results of the present study demonstrated that in vivo infection of VRSA causes alteration of oxidant-antioxidant status in spleen, as evidenced by enhanced NO, MPO, MDA, PC, and GSSG level and decreased GSH level and also SOD, CAT, GPx, GR, and GST activity.

CMC-EDBE-FA nanoparticles were prepared by the carboxylic group (–COOH) of folic acid and –COOH group of functionalized carboxymethyl chitosan connected through the end-amino groups hydrophilic spacer, 2,2′-(ethylenedioxy)bis(ethylamine). It is well known that carboxymethyl chitosan is easily soluble in water but folic acid is much less soluble in water. When carboxymethyl chitosan is connected by folic acid through a spacer, carboxymethyl chitosan may act as a hydrophilic part and folic acid as a hydrophobic part.

The results of our study demonstrates that nanoconjugated vancomycin eliminates the bacterial infection and recovers the spleen from infection (Figures 1 and 2), indicating its beneficial role against VRSA infection. It is evident from our study that VRSA infection in mice is associated with enhanced nitrate generation, MPO activity, MDA level, PC level, and GSSG level and decreased GSH level and as well as decreased enzymatic antioxidant (SOD, CAT, GPx, GR, and GST) activity in spleen, which are ameliorated by treatment of nanoconjugated vancomycin (Figures 3–7). Moreover DNA damage assessed by DPA assay due to VRSA infection was also observed in spleen, which is protected by treatment of nanoconjugated vancomycin (Figure 8). 

In this study, significant elevation of nitrate level and MPO activity in spleen was observed in VRSA-infected mice, which were decreased in nanoconjugated-vancomycin-treated group. Treatment of nanoconjugated vancomycin to VRSA-infected mice decreased NO level and MPO activity significantly in spleen (Figure 3). Nitric oxide (NO) is a free radical synthesized by nitric oxide synthase (NOS). NOS is composed of two identical monomers with molecular weights ranging from 130 to 160 kDa [23]. Our previous study showed that nitric oxide synthesis in lymphocytes as well as its release in serum is high during VSSA and VRSA infection, which can be related to an alteration in oxidant-antioxidant potential [16]. Thus, higher level of nitrite by VRSA infection may be due to high production of free radicals. Nanoconjugated vancomycin plays the role of antioxidant to prevent the nitrate generation maybe through the inhibition of inducible nitric oxide synthase (iNOS) expression [24]. Hypochlorous acid (HOCl) is generated in the presence of myeloperoxidase and initiates the deactivation of antiproteases and the activation of latent proteases and leads to the cellular damage [25]. In this study, nanoconjugated vancomycin inhibited the myeloperoxidase activity which was increased due to VRSA infection, suggesting a protective role of nanoconjugated vancomycin (Figure 3). These results suggest that the cellular antioxidants level either was reached in a higher concentration to exert antioxidant effects or scavenged the free radical produced by the myeloperoxidase [26]. Thus, in addition to the antioxidant system, nanoconjugated vancomycin may indirectly protect spleen from VRSA-infection-induced oxidative damage. Thus, free radical depletion by the antioxidant agents seems to be beneficial for preventing the damage of lipid and protein.

In this study, significant elevation of malondialdehyde (MDA) and protein carbonyl level was observed in spleen of VRSA-infected mice, whereas treatment of nanoconjugated vancomycin in VRSA-infected mice decreased lipid peroxidation and protein oxidation significantly in lymphocytes (Figure 4). It may be due to the generation of free radicals (mainly NO) which may react with protein in addition to lipids. Lipid peroxidation is known to disturb the integrity of cellular membranes, leading to the leakage of cytoplasmic enzymes [27]. Protein carbonyls formation has been indicated to be an earlier marker of protein oxidation. Oxidation of protein may be due to either excessive oxidation of proteins or decreased capacity to clean up oxidative damaged proteins. Oxidative modification of proteins may lead to the structural alteration and functional inactivation of many enzyme proteins [28], as evidenced by the decreased activity of different antioxidant enzymes like SOD, CAT, GPx, GR, and GST. 

Imbalance between the generation of reactive oxygen species (ROS) and the antioxidant system causes oxidative stress. Glutathione, an important cellular reductant, is involved in protection against free radicals, peroxides and toxic compounds in cellular systems [29]. In the present study, the reduced glutathione level was significantly decreased in spleen of VRSA-infected mice, whereas treatment of nanoconjugated vancomycin in VRSA-infected mice increased the GSH level (Figure 5). In this study, it was observed that oxidized glutathione level was increased in spleen of VRSA-infected mice, which was ameliorated due to nanoconjugated vancomycin treatment (Figure 5). The decreased GSH levels represent its increased utilization due to VSSA and VRSA infection. On the other hand, decreasing GSH level may be due to increasing level of lipid oxidation products which may be associated with less availability of NADPH required for the activity of glutathione reductase (GR) to transform GSSG to GSH due to the increasing production of ROS in form of NO [30]. In our present study, the increasing levels of GSSG and decreasing GR activity (Figure 7) due to VRSA infection may support the explanation.

Antioxidant enzymes are considered to be a primary defense that prevents biological macromolecules from oxidative damage. SOD rapidly dismutates superoxide anion (O_2_^.−^) to less dangerous H_2_O_2_, which is further degraded by CAT and GPx to water and oxygen [31]. The results of the present study showed a significant fall in SOD and CAT activities in spleen of VRSA-infected group, whereas treatment of nanoconjugated vancomycin in VRSA-infected mice significantly increased the SOD and CAT activity (Figure 6). SOD, dismutates O_2_^.−^ and the same in turn is a potent inhibitor of CAT [32]. The depletion in SOD activity was maybe due to disposing off the free radicals, produced due to VSSA and VRSA infection. Beside this, during infection, H_2_O_2_ produced by dismutation of superoxide anion may have been efficiently converted to O_2_ by CAT and the enzyme activities showed a marked reduction. The depletion of antioxidant enzyme activity was maybe due to inactivation of the enzyme proteins by VSSA and VRSA-infection-induced NO generation, depletion of the enzyme substrates, and/or downregulation of transcription and translation processes.

GPx works nonspecifically to scavenge and decompose excess hydroperoxides including H_2_O_2_, which may be prevalent under oxidative stress [33]. Glutathione-s-transferase (GST) mainly detoxifies electrophilic compounds [34] and has a well-established role in protecting cells from mutagens and carcinogens as a free radical scavenger along with glutathione. In the present study, the significant decreasing of GSH level and GSH-dependent enzymes, that is, GPx, GR, and GST (Figure 7), in VRSA infection may be due to increased utilization to scavenge the free radical generation. The results of the present study showed a significant fall of GPx, GR, and GST activities in spleen of VRSA-infected group, whereas treatment of nanoconjugated vancomycin in VRSA-infected mice significantly increased the GPx, GR, and GST activity in spleen (Figure 7). In the present study, it was observed that MDA level (Figure 4) and DNA fragmentation (Figure 8) were significantly elevated in spleen due to VRSA infection. This elevated MDA level decreases GSH level (Figure 5) and SOD activity (Figure 6), which may be associated with DNA fragmentation. In this study, it was observed that DNA fragmentation increased in VRSA-infected spleen, which was brought back near to control due to nanoconjugated vancomycin treatment.

In conclusion, the study showed here that, spleen is susceptible to VRSA infection through the increased production of nitric oxide which leads to decreased antioxidant status in spleen and nanoconjugated vancomycin protects the spleen from such infection by decreasing free radical generation, lipid, and protein damage, and also by increasing the antioxidant status. Hence, the nanoconjugated vancomycin can be used as a potent free radical scavenger antioxidative product and can be used as a potential therapeutic agent against staphylococcal infection. 

## Abbreviations

CAT: Catalase
CFU: Colony formation unit
CMC: Carboxymethyl chitosan
CMC-EDBE-FA: Carboxymethyl chitosan-2,2′-(ethylenedioxy)bis(ethylamine)-folate
CS: Chitosan
DNA: Deoxyribonucleic acid
DPA: Diphenylamine
DPX: Dibutyl phthalate xylol
DTNB: 5′,5′-dithio(bis)-2-nitrobenzoic acid
EDTA: Ethylene diamine tetra acetate
GPx: Glutathione peroxidase
GR: Glutathione reductase
GSSG: Oxidized glutathione
GSH: Reduced glutathione
GST: Glutathione-s-transferase
H_2_O_2_: Hydrogen peroxide
MDA: Malondialdehyde
MPO: Myeloperoxidase
MRSA: Methicillin-resistant *Staphylococcus aureus *
NADPH: Nicotinamide adenine dinucleotide phosphate
NO: Nitric oxide
PC: Protein carbonyls
ROS: Reactive oxygen species
SDS: Sodium dodecyl sulfate
SOD: Superoxide dismutase
SSA: Sulfosalicylic acid
TBA: Thiobutiric acid
TBARS: Thiobutiric acid reactive substance
TCA: Trichloroacetic acid
VRSA: Vancomycin-resistant *Staphylococcus aureus*.
